# Utilisation and Off-Label Prescriptions of Respiratory Drugs in Children

**DOI:** 10.1371/journal.pone.0105110

**Published:** 2014-09-02

**Authors:** Sven Schmiedl, Rainald Fischer, Luisa Ibáñez, Joan Fortuny, Olaf H. Klungel, Robert Reynolds, Roman Gerlach, Martin Tauscher, Petra Thürmann, Joerg Hasford, Marietta Rottenkolber

**Affiliations:** 1 Department of Clinical Pharmacology, School of Medicine, Faculty of Health, Witten/Herdecke University, Witten, Germany; 2 Philipp Klee-Institute for Clinical Pharmacology, HELIOS Clinic Wuppertal, Wuppertal, Germany; 3 Pneumologische Praxis München-Pasing, Munich, Germany; 4 Fundació Institut Català de Farmacologia, Hospital Universitari Vall d'Hebron, Barcelona, Spain; 5 Departament de Farmacologia, Terapèutica i Toxicologia, Universitat Autònoma de Barcelona, Barcelona, Spain; 6 Novartis Farmaceutica S.A., Barcelona, Spain; 7 Utrecht Institute for Pharmaceutical Sciences, Division Pharmacoepidemiology and Clinical Pharmacology, Utrecht University, Utrecht, the Netherlands; 8 Epidemiology, Pfizer, New York, New York, United States of America; 9 National Association of Statutory Health Insurance Physicians of Bavaria, Munich, Germany; 10 Institute for Medical Information Sciences, Biometry, and Epidemiology, Ludwig-Maximilians-Universitaet, Munich, Germany; Nottingham University, United Kingdom

## Abstract

Respiratory drugs are widely used in children to treat labeled and non-labeled indications but only some data are available quantifying comprehensively off-label usage. Thus, we aim to analyse drug utilisation and off-label prescribing of respiratory drugs focusing on age- and indication-related off-label use. Patients aged ≤18 years documented in the Bavarian Association of Statutory Health Insurance Physicians database (approx. 2 million children) between 2004 and 2008 were included in our study. Annual period prevalence rates (PPRs) per 10,000 children and the proportion of age- and indication-related off-label prescriptions were calculated and stratified by age and gender. Within the study period, highest PPRs were found for the fixed combination of clenbuterol/ambroxol (between 374–575 per 10,000 children) and the inhaled short acting beta-2-agonist salbutamol (between 378–527 per 10,000 children). Highest relative PPR increase was found for oral salbutamol (approx. 39-fold) whereas the most distinct decrease was found for oral long-acting beta-2-agonist clenbuterol (−97%). Compound classes most frequently involved in off-label prescribing were inhaled bronchodilative compounds (91,402; 37.3%) and oral beta-2-agonists (26,850; 22.5%). The highest absolute number of off-label prescriptions were found for inhaled salbutamol (n = 67,084; 42.0%) and oral clenbuterol/ambroxol (fixed combination, n = 18,897; 20.7%). Off-label prescribing due to indication was of much greater relevance than age-related off-label use. Most frequently, bronchodilative compounds were used off-label to treat respiratory tract infections. Highest off-label prescription rates were found in the youngest patients without relevant gender-related differences. Off-label prescribing of respiratory drugs is common especially in young children. Bronchodilative drugs were most frequently used off-label for treating acute bronchitis or upper respiratory tract infections underlining the essential need for a more rational prescribing in this area.

## Introduction

Respiratory drugs are frequently prescribed to paediatric patients for a wide range of airway diseases but most of these drugs are only approved for asthma and COPD from a particular age onwards [1], [2]. In young children, diagnosing asthma is difficult due to general limitations (e.g. ability to follow instructions for lung function measurements) which might contribute to a low fraction of patients with lung function testing and an under-diagnosis of asthma [3]. In addition, respiratory drugs are frequently used for symptomatic improvement of airway diseases (e.g. acute respiratory infections) or are prescribed as a diagnostic instrument to confirm a diagnosis of asthma [1], [2]. All these reasons contribute to a high fraction of children receiving respiratory medication as off-label treatment [1], [2], a factor which has been reported as a risk for adverse drug reactions [4]–[6].

Whereas some data are available about anti-asthmatic drug utilisation in children [7], [8], the extent of off-label usage for these compounds has been quantified only in few studies [1], [2], [9]. Furthermore, generalizability of off-label results is limited due to e.g. national drug market characteristics and differing age groups of patients included in these studies [1], [9]. In addition, some other aspects as for example time trends in off-label prescriptions or gender-related aspects have not been analysed in detail in these studies.

Thus, we aimed to analyse drug utilisation and indication- and age-related off-label use for respiratory drugs in children.

## Methods

### Database and study population

This study was performed using the Bavarian Association of Statutory Health Insurance Physicians database which covers approximately 2 million insured children aged ≤18 years (85% of the Bavarian paediatric population) excluding those with a private insurance [10]. All diagnoses of general practitioners and specialists were documented and a prescription was recorded in the database only if it was prescribed and filled at the pharmacy. Diagnoses and drugs were coded according to the International Classification of Diseases codes (ICD-10-GM) and the Anatomical Therapeutic Chemical (ATC-) classification, respectively [11], [12]. Every child (≤18 years) receiving at least one prescription of respiratory drugs as stated in table 1 between 2004 and 2008 were included in the study. Analyses were restricted to drugs with an annual period prevalence rate (PPR) for the year 2008 of at least 0.1 per 10,000 children. All analyses were done using completely anonymised data only. The German law and the professional code of conduct for physicians do not ask for an ethical review for research with anonymised data.

**Table 1:** Age restrictions and indications for selected respiratory drug classes.

Compound class	Compound	ATC-code*	Age restriction	Approved indication(s)
**Inhaled SABA**	Salbutamol	R03AC02	None	Asthma, chronic obstructive bronchitis, pulmonary emphysema with reversible obstruction, prophylaxis of allergic asthma and exercise-induced asthma
	Fenoterol	R03AC04	≥4 years	Asthma, chronic obstructive bronchitis, pulmonary emphysema with reversible obstruction, prophylaxis of allergic asthma and exercise-induced asthma
	Terbutaline	R03AC03	≥5 years	Asthma, chronic obstructive bronchitis, pulmonary emphysema with reversible obstruction
**Inhaled SABA combination**	Ipratropium/Fenoterol (fixed combination)	R03AK03	None	Asthma, COPD
	Reproterol/CGA (fixed combination)	R03AK05	None	Asthma
**Inhaled LABA**	Salmeterol	R03AC12	≥4 years	Asthma, COPD
	Formoterol	R03AC13	≥6 years	Asthma, COPD
**Inhaled LABA/ICS**	Salmeterol/Fluticasone (fixed combination)	R03AK06	≥4 years	Asthma, COPD
	Formoterol/Beclomethasone (fixed combination)	R03AK27	≥6 years	Asthma
	Formoterol/Budesonide (fixed combination)	R03AK28	≥6 years	Asthma, COPD
**Inhaled SAMA**	Ipratropium	R03BB01	None	Asthma, COPD
**Inhaled LAMA**	Tiotropium	R03BB04	≥18 years	COPD
**ICS**	Budesonide	R03BA02	None	Respiratory diseases (inclusive asthma and COPD) requiring ICS
	Beclomethasone	R03BA01	None	Respiratory diseases (inclusive asthma and COPD) requiring ICS
	Fluticasone	R03BA05	≥4 years	Asthma, COPD
	Ciclesonide	R03BA08	≥12 years	Asthma
**Oral B2A**	Salbutamol	R03CC02	None	Obstructive respiratory diseases, asthma, COPD, pulmonary emphysema
	Terbutaline	R03CC03	None	Obstructive respiratory diseases, asthma, COPD, pulmonary emphysema
	Tulobuterol	R03CC11	≥1year	Obstructive respiratory diseases, asthma, COPD, pulmonary emphysema
	Clenbuterol	R03CC13	None	Asthma, asthmatic bronchitis, chronic bronchitis, pulmonary emphysema
**Oral B2A combinations**	Clenbuterol/Ambroxol	R03CC63	None	Acute and chronic bronchitis, pulmonary emphysema, asthma
**Others**	Theophylline	R03DA04	≥1year	Asthma, COPD
	Montelukast	R03DC03	≥1 year	Asthma, prophylaxis of exercise-induced asthma
	Cromoglicic acid	R03BC01	≥2 years	Asthma

SABA: Short-acting beta-2-agonist, CGA: Cromoglicic acid, LABA: Long-acting beta-2-agonist, ICS: Inhaled corticosteroid, SAMA: Short-acting muscarinic antagonist, LAMA: Long-acting muscarinic antagonist, B2A: Beta-2-agonist. (*German version available at http://www.dimdi.de/static/de/amg/atcddd/index.htm).

### Off-label analysis

For compounds stated in table 1, off-label analysis based on patient's age and indication was performed. The lower approved age and the approved indication were collected using the official summary of product characteristics (SPC) [13], [14] and the Pharmaceutical Index for Germany [15] for the years 2004 and 2008 (beginning and end of the study period, table 1). If more than one age restriction existed for different devices or different years within the study period, we used the lowest age restriction for the respective ATC code. To analyse off-label prescriptions by indication, the widest definition was used if more than one definition for indications existed (table 1).

### Statistical analysis

Annual period prevalence rates (PPRs) were calculated using the number of children with at least one prescription of interest during the year of interest (numerator) divided by the total number of children living in Bavaria at the end of the year (December, 31; denominator), based on the data of the Bavarian State Office for Statistics and Data Processing [16]. Under the assumption of equal age- and gender-distribution of children in the statutory and private health insurance, we used a correction factor (0.85) considering the statutory health insurance coverage of 85% of the total Bavarian population. Annual PPRs were calculated and stratifications by age (one-year age groups) and gender were performed.

Off-label prescriptions were analysed as proportion and stratifications by type of off-label prescriptions (‘age’, ‘indication’, and ‘age&indication’), patient's age (one-year age groups) and gender were performed. All analyses were performed using IBM SPSS Statistics Version 20.0 and GNU R Version 3.0.1 (http://www.r-project.org/).

## Results

### Drug utilization

**Figure 1:**
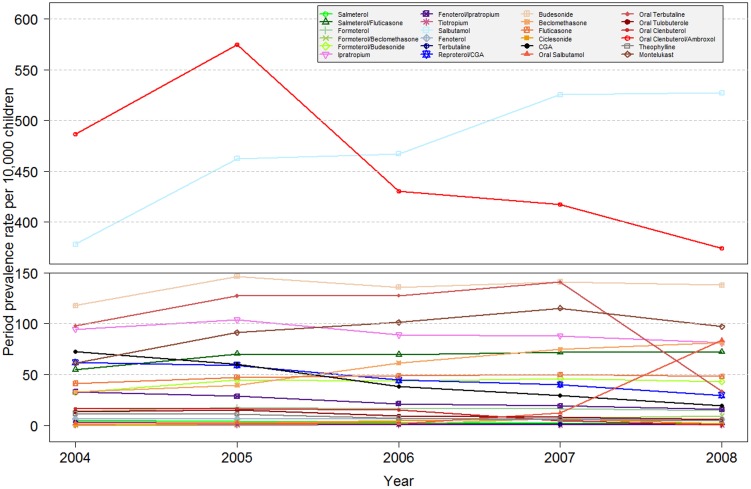
Annual period prevalence rates per 10,000 children (≤18 years) between 2004 and 2008.

Within the study period, highest annual PPRs were found for the fixed combination of oral clenbuterol/ambroxol (between 374–575 per 10,000 children) and the inhaled short acting beta-2-agonist salbutamol (between 378–527 per 10,000 children). By comparing PPRs of 2004 and 2008, the highest absolute PPR increase was found for inhaled salbutamol (+149 per 10,000 children) whereas for clenbuterol/ambroxol, the most pronounced decrease was found (−113 per 10,000 children). Regarding relative PPR changes, highest increases were found for oral salbutamol (approx. 39-fold) and the fixed combination beclomethasone (approx. 2.5-fold) whereas the most distinct decrease was found for oral clenbuterol (−97%) and inhaled terbutaline (−77%, figure 1).

For 2008, age-related PPR patterns were similar for both genders but for most compounds, PPRs were higher for boys compared to girls. In children aged <6 years old, inhaled salbutamol and the fixed combination oral clenbuterol/ambroxol were the most frequently prescribed compounds whereas in adolescents (15–18 years), highest PPRs were found for salbutamol and budesonide (Table S1, S2).

### Off-label prescriptions

The extent of off-label prescriptions related to the three types of off-label prescriptions ‘age’, ‘indication’ or ‘age&indication’ is shown in table 2 for the year 2008. The highest absolute number of off-label prescriptions including these three types of off-label prescribing were found for inhaled bronchodilative drugs (i.e. short-acting beta-2-agonist (SABA), long-acting beta-2-agonist (LABA), short-acting muscarinic antagonist (SAMA), and long-acting muscarinic antagonist (LAMA), including fixed combinations) with n = 91,402 (37.3% of all inhaled bronchodilative drugs) followed by oral beta-2-agonists (including fixed combinations) with n = 26,850 (22.5%). Regarding single compounds, inhaled salbutamol (n = 67,084; 42.0%) and oral clenbuterol/ambroxol (fixed combination, n = 18,897; 20.7%) were most frequently prescribed off-label. In most of these patients, off-label prescriptions due to “indication only” were present (inhaled bronchodilative drugs: 90,443 [99.0% of all off-label prescriptions], inhaled salbutamol 67,084 [100%], oral beta-2-agonists (including fixed combinations): 26,812 [99.9%], and oral clenbuterol/ambroxol (fixed combination): 18,897 [100%]; table 2).

**Table 2:** Number and proportion of off-label prescriptions stratified by off-label type (year 2008).

Compound class	Compound	All prescriptions (n)	Off-label overall (n,%)	Off-label due to age only (n)	Off-label due to indication only (n)	Off-label due to age and indication (n)
**Inhaled SABA**	**Salbutamol**	159,655	67,084 (42.0%)	0	67,084	0
	**Fenoterol**	1,452	383 (26.4%)	6	367	10
	**Terbutaline**	184	34 (18.5%)	0	33	1
**Inhaled SABA combination**	**Fenoterol/Ipratropium (fixed combination)**	3,998	1,722 (43.1%)	0	1,722	0
	**Reproterol/CGA (fixed combination)**	8,729	2,538 (29.1%)	0	2,538	0
**Inhaled SABA (incl. combination) Total**		174,018	71,761 (41.2%)	6	71,744	11
**Inhaled LABA**	**Salmeterol**	522	86 (16.5%)	9	75	2
	**Formoterol**	4,931	839 (17.0%)	88	730	21
**Inhaled LABA/ICS**	**Salmeterol/Fluticasone (fixed combination)**	27,600	4,278 (15.5%)	368	3,734	176
	**Formoterol/Beclomethasone (fixed combination)**	2,515	848 (33.7%)	11	827	10
	**Formoterol/Budesonide (fixed combination)**	13,833	2,584 (18.7%)	113	2,412	59
**Inhaled LABA (incl. combination) Total**		49,401	8,635 (17.5%)	589	7,778	268
**SAMA**	**Ipratropium**	21,822	10,910 (50.0%)	0	10,910	0
**LAMA**	**Tiotropium**	97	96 (99.0%)	9	11	76
**Muscarinic antagonists (SAMA & LAMA) Total**		21,919	11,006 (50.2%)	9	10,921	76
**Inhaled bronchodilative drugs (SABA, LABA, SAMA, LAMA [incl. combination]) Total**		**245,338**	**91,402 (37.3%)**	**604**	**90,443**	**355**
**ICS**	**Budesonide**	42,067	3,166 (7.5%)	0	3,166	0
	**Beclomethasone**	24,185	1,922 (7.9%)	0	1,922	0
	**Fluticasone**	17,097	5,360 (31.4%)	1,917	2,370	1,073
	**Ciclesonide**	326	110 (33.7%)	35	60	15
**ICS total**		83,675	10,558 (12.6%)	1,952	7,518	1,088
**Oral B2A**	**Salbutamol**	19,475	5,544 (28.5%)	0	5,544	0
	**Terbutaline**	6,940	2,012 (29.0%)	0	2,012	0
	**Tulobuterol**	1,201	330 (27.5%)	32	292	6
	**Clenbuterol**	113	67 (59.3%)	0	67	0
**Oral B2A combination**	**Clenbuterol/Ambroxol (fixed combination)**	91,385	18,897 (20.7%)	0	18,897	0
**Oral B2A (incl. combination) total**		119,114	26,850 (22.5%)	32	26,812	6
**Others**	**Theophylline**	1,184	451 (38.1%)	7	419	25
	**Montelukast**	33,501	12,826 (38.3%)	304	11,831	691
	**Cromoglicic acid**	5,087	3,247 (63.8%)	110	2,724	413
**Others Total**		39,772	16,524 (41.5%)	421	14,974	1,129
**All drugs Total**		**487,899**	**145,334 (29.8%)**	**3,009**	**139,747**	**2,578**

SABA: Short-acting beta-2-agonist, CGA: Cromoglicic acid, LABA: Long-acting beta-2-agonist, ICS: Inhaled corticosteroid, SAMA: Short-acting muscarinic antagonist, LAMA: Long-acting muscarinic antagonist, B2A: Beta-2-agonist.

By analyzing off-label indications in detail (combined analysis of off-label prescriptions due to ‘indication’ and ‘age&indication’; table 3), we found that inhaled salbutamol was most frequently used off-label for treating acute bronchitis (n = 29,989, 44.7% of all off-label prescriptions due to ‘indication’ and ‘age&indication’) and acute upper respiratory infections (n = 23,827, 35.5% [multiple counting of off-label indications]). Similarly, oral clenbuterol/ambroxol (fixed combination) was most frequently prescribed off-label for treating acute upper respiratory infections (n = 9,131, 48.3%). For the compound classes most frequently used off-label (i.e. inhaled bronchodilative drugs and oral beta-2-agonists [including fixed combinations]), acute bronchitis and/or acute upper respiratory tract infections were the most common off-label indications (Table S3).

**Table 3:** Number and proportion of the three most frequent off-label indications for drugs with at least 5,000 prescriptions (year 2008, multiple counting of off-label indications per prescription).

Compound class	Compound	All prescriptions (n)	Off-label due to ‘indication’ or ‘age&indication’ (n,% of all prescriptions)	Three most frequent off-label indications* (n, % of all off-label prescriptions due to ‘indication’ and ‘age&indication’)
**Inhaled SABA**	**Salbutamol**	159,655	67,084 (42.0%)	Acute bronchitis: 29,989 (44.7%), Acute upper respiratory infections: 23,827 (35.5%), Other diseases of upper respiratory tract: 13,267 (19.8%)
**Inhaled SABA combination**	**Reproterol/CGA (fixed combination)**	8,729	2,538 (29.1%)	Other disease of upper respiratory tract: 999 (39.4%), Acute upper respiratory tract infections: 402 (15.8%), Bronchitis nec: 259 (10.2%)
**Inhaled LABA/ICS**	**Salmeterol/Fluticasone (fixed combination)**	27,600	3,910 (14.2%)	Other diseases of upper respiratory tract: 1,066 (27.3%), Acute bronchitis: 751 (19.2%), Acute upper respiratory infections: 679 (17.4%)
	**Formoterol/Budesonide (fixed combination)**	13,833	2,471 (17.9%)	Other diseases of upper respiratory tract: 621 (25.1%), Acute upper respiratory infections: 456 (18.5%), Bronchitis nec: 428 (17.3%)
**Inhaled SAMA**	**Ipratropium**	21,822	10,910 (50.0%)	Acute bronchitis: 5,779 (53.0%), Acute upper respiratory infections: 4,124 (37.8%), Bronchitis nec: 2,033 (18.6%)
**ICS**	**Fluticasone**	17,097	3,443 (20.1%)	Acute bronchitis: 1,096 (31.8%), Acute upper respiratory infections: 977 (28.4%), Other diseases of upper respiratory tract: 847 (24.6%)
**Oral B2A**	**Oral Salbutamol**	19,475	5,544 (28.5%)	Acute upper respiratory infections: 2,431 (43.8%), Bronchitis nec: 2,020 (36.4%), Other diseases of upper respiratory tract: 1,001 (18.1%)
	**Oral Terbutaline**	6,940	2,012 (29.0%)	Acute upper respiratory infections: 880 (43.7%), Bronchitis nec: 773 (38.4%), Other diseases of upper respiratory tract: 348 (17.3%)
**Oral B2A combination**	**Oral Clenbuterol/Ambroxol (fixed combination)**	91,385	18,897 (20.7%)	Acute upper respiratory infections: 9,131 (48.3%), Other diseases of upper respiratory tract: 2,767 (14.6%), Other respiratory diseases: 2,228 (11.8%)
**Others**	**Montelukast**	33,501	12,522 (37.4%)	Acute bronchitis: 3,868 (30.9%), Acute upper respiratory infections: 3,850 (30.7%), Other diseases of upper respiratory tract: 3,014 (24.1%)
	**Cromoglicic acid**	5,087	3,137 (61.7%)	Acute upper respiratory infections: 1,151 (36.7%), Acute bronchitis: 1,101 (35.1%), Other diseases of upper respiratory tract: 789 (25.2%)

SABA: Short-acting beta-2-agonist, CGA: Cromoglicic acid, LABA: Long-acting beta-2-agonist, ICS: Inhaled corticosteroid, SAMA: Short-acting muscarinic antagonist, B2A: Beta-2-agonist, nec: not elsewhere classified.

*Exclusive missing indications.

Regarding age, we observed the highest proportion of off-label prescriptions in youngest children aged under 6 years. For some compounds (e.g. formoterol) we found highest off-label prescriptions in youngest children and adolescents (u-shape; Table S4). We did not observe relevant differences in off label prescriptions between male and female children (Table S4). Focussing on changes over time (2004 versus 2008), the increase in the absolute number of off-label prescriptions was highest for inhaled (n = 21,841; +48.3%) and oral salbutamol (n = 5,422; +4,444.3%) whereas the highest decrease was found for CGA (n = −10,195; −75.8%) and oral clenbuterol/ambroxol (n = −6,196; −24.7%). In contrast, we found only small changes (less than 10%) in the proportion of off-label prescriptions for most compounds comparing 2004 and 2008 indicating that observed changes in absolute numbers of off-label prescriptions are mainly attributable to changes in absolute numbers of total prescriptions (Table S5).

## Discussion

By analysing prescription patterns of respiratory drugs, we found highest PPRs for the fixed combination of clenbuterol/ambroxol and inhaled salbutamol. In our study, highest absolute numbers of off-label prescriptions were found for bronchodilative compounds including the most frequently prescribed drugs (i.e. inhaled salbutamol and the fixed combination of oral clenbuterol/ambroxol). For most compounds, off-label prescribing was mainly due to indication for treating respiratory tract infections.

### Drug utilisation

Similar to our results, there are several studies reporting SABA and ICS as most prevalent respiratory drugs used in children [7], [8], [17]. Nevertheless, there is some inter-country variation as reported by Bianchi et al. [7]. Whereas SABA is the most prescribed anti-asthmatic drug class in e.g. Denmark and the USA, in Italy inhaled corticosteroids is the most frequently prescribed drug class. Despite similar results, a comparison of these studies with our results is limited due to some methodological aspects (e.g. differences in age groups and source of prescription data). Furthermore, national specialities in drug markets (i.e. national drug approval for “older” compounds) and historically grown, specific national prescription behaviour might have contributed to some differences. In Germany, for example, a fixed combination of clenbuterol/ambroxol has been widely used whereas in other countries, no comparable fixed combination drug is available.

There are few studies reporting trends in asthma medication prescriptions [1], [17]. Whereas Elkout et al. reported the fraction of patients aged less than 19 years of age treated with a particular drug class [17], Baiardi et al. presented the number of prescriptions for eleven compounds representing 90% of R03 (according to ATC) prescriptions given to children aged between 0 and 14 years of age [1]. Despite these methodological differences compared to our study reporting PPR, some issues are worth to be mentioned. Baiardi et al. [1] reported a stable number of inhaled salbutamol prescriptions whereas in our study, we found an increase in inhaled and oral salbutamol prescriptions which might correspond to a decreased prescriptions for the fixed combination of oral (long-acting beta-2-agonist) clenbuterol/ambroxol and oral terbutaline.

### Off-label prescriptions

In our study, highest absolute numbers of off-label prescriptions were found for inhaled salbutamol and the fixed combination of oral clenbuterol/ambroxol. In most cases, off-label prescriptions were made to treat acute respiratory tract infections. In general, we found that a much higher proportion of off-label prescriptions were due to indication than due to age. This has also been reported by Baiardi et al. [1] whereas Sen et al. [9] did not report comprehensively age-related off-label usage and did primarily focus on indication-related off-label use.

Comparing our results for indication related off-label prescriptions with other studies, we found similar proportions for most compounds (Table S6). Nevertheless, some methodological issues will limit transferability of results. In addition to the issues mentioned already for the analyses conducted by Baiardi et al. [1], Sen et al. [9] did use general practice databases whereas in our study, prescriptions made by specialists are included too. Furthermore, we used the widest definition stated in the SPC for a specific compound whereas Baiardi et al. [1] did use more restrictive definitions of indications leading to a higher rates of off-label prescriptions for some compounds (Table S6). Since Zuidgeest et al. [2] did not report compound specific off-label rates, we abstained from presenting and discussing these results in detail.

As described by Baiardi et al. [1], respiratory tract infections and bronchiolitis were frequent off-label diagnoses in our study, too. Apart from formal implications of using drugs outside the approved indications, these results may underline a somewhat irrational prescribing which has been criticized before [18], [19]. According to guidelines, systematic reviews, and meta-analyses, neither beta-2-agonists nor inhaled corticosteroids should be routinely recommended as treatment options for these indications due to lacking efficacy [20]–[23]. Of course in some patients, bronchodilators may lead to a transient clinical improvement but this should be weighed against potential adverse effects and the fact that most children will not benefit [20]. Nevertheless, as shown by de Brasi et al. [19] and Ochoa Sangrador et al. [24], there is a relevant overuse of both compounds which has been attributed to e.g. physicians' recognition of disease severity, personal reassurance, and parental pressure [19]. On the other hand, by developing and implementing clinical guidelines, a more rational prescribing leading to less overtreatment seems reachable [25], [26].

### Limitations and Strengths

As for all observational studies, there are few limitations worth to be mentioned. First, as in most claims data analyses, we were not able to include clinical data (e.g. lung function parameter) and thus, we did focus on drug prescription instead of analysing patients in detail comparing on- and off-label users (as already done [27]). Second, we did analyse off-label treatment based on a compound- and not on a device-level using widest restrictions (age and/or indication) if different age restrictions or indications were mentioned in the respective summary of product characteristics. This approach will lead to an underestimation of off-label prescriptions for some compounds (e.g. formoterol, fixed combination of salmeterol/fluticasone) whereas for the majority of drugs or drug classes, all available devices have the same age restriction and labeled indication. Third, there are some uncertainties in matching specific ICD-codes needed for analyzing databases and indications stated in the SPC in particular when general terms have been used in the SPC. This might have influenced the number of calculated off-label prescriptions. Nevertheless, most of the terms used for defining off-label usage are comparable to other publications [1]. Fourth, within on-label prescriptions we did not discriminate between different compound classes regarding their efficiency. For example, asthma is a labeled indication for ipratropium but the role of anticholinergic compounds has been critically discussed in particular for asthmatic children [28]. Furthermore, inhaled SABA is the recommended reliever treatment whereas inhaled anticholinergics are considered only as alternative treatments according to the guidelines [29]. Fifth, since we use a statutory health insurance database, children with a private health insurance were not included. Hence, a bias due to socioeconomic status can not be excluded in our study. But one has to keep in mind that the database used covers with 85% the majority of the children in Bavaria.

Besides a few limitations, there are also some strengths of our study. First of all, we did use a large database with a good population coverage (85%) covering 2.0 million children. Second, not only data from general practitioners but also from specialists were included in our study. Third, since we did analyse a time period and did not only perform a cross-sectional analysis, we are able to quantify time trends in off-label prescriptions, which (to the best our knowledge) has not been performed before for children receiving respiratory medication.

## Conclusion

In our study analysing respiratory drugs, we found highest PPRs for inhaled salbutamol and the fixed combination of oral clenbuterol/ambroxol. Off-label prescribing of respiratory drugs is common especially in young children. Bronchodilative drugs were most frequently used off-label for treating acute bronchitis or upper respiratory tract infections underlining the essential need for a more rational prescribing in this area.

## Supporting Information

Table S1 **Period prevalence rates for boys stratified by age for the year 2008.** SABA: Short-acting beta-2-agonist, CGA: Cromoglicic Acid, LABA: Long-acting beta-2-agonist, ICS: Inhaled corticosteroid, SAMA: Short-acting muscarinic antagonist, LAMA: Long-acting muscarinic antagonist, B2A: Beta-2-agonist.
(DOC)

Table S2 **Period prevalence rates for girls stratified by age for the year 2008.** SABA: Short-acting beta-2-agonist, CGA: Cromoglicic Acid, LABA: Long-acting beta-2-agonist, ICS: Inhaled corticosteroid, SAMA: Short-acting muscarinic antagonist, LAMA: Long-acting muscarinic antagonist, B2A: Beta-2-agonist.
(DOC)

Table S3 **Number and proportion of off-label indications (year 2008, multiple counting of off-label indications per prescription).** SABA: Short-acting beta-2-agonist, CGA: Cromoglicic Acid, LABA: Long-acting beta-2-agonist, ICS: Inhaled corticosteroid, SAMA: Short-acting muscarinic antagonist, LAMA: Long-acting muscarinic antagonist, B2A: Beta-2-agonist, nec: not elsewhere classified, n.a.: not applicable (i.e. labeled diagnoses).
(DOC)

Table S4 **Number and proportion of off-label prescriptions stratified by off-label type, gender, and age group (year 2008).** SABA: Short-acting beta-2-agonist, CGA: Cromoglicic Acid, LABA: Long-acting beta-2-agonist, ICS: Inhaled corticosteroid, SAMA: Short-acting muscarinic antagonist, LAMA: Long-acting muscarinic antagonist, B2A: Beta-2-agonist.
(DOC)

Table S5 **Number and proportion of off-label prescriptions for the years 2004 to 2008.** SABA: Short-acting beta-2-agonist, CGA: Cromoglicic Acid, LABA: Long-acting beta-2-agonist, ICS: Inhaled corticosteroid, SAMA: Short-acting muscarinic antagonist, LAMA: Long-acting muscarinic antagonist, B2A: Beta-2-agonist, n.a.: not applicable.
(DOC)

Table S6 **Comparison of the proportion of off-label usage due to indication in several European countries.** SABA: Short-acting beta-2-agonist, LABA: Long-acting beta-2-agonist, ICS: Inhaled corticosteroid, SAMA: Short-acting muscarinic antagonist.
(DOC)
