# Supplementary material for: Utilisation and Off-Label Prescriptions of Respiratory Drugs in Children
Source: PLoS One. 2014 Sep 2;9(9):e105110. doi: 10.1371/journal.pone.0105110 (PMC4152124; doi:10.1371/journal.pone.0105110)
Supplement: Table S1 — Period prevalence rates for boys stratified by age for the year 2008. SABA: Short-acting beta-2-agonist, CGA: Cromoglicic Acid, LABA: Long-acting beta-2-agonist, ICS: Inhaled corticosteroid, SAMA: Short-acting muscarinic antagonist, LAMA: Long-acting muscarinic antagonist, B2A: Beta-2-agonist. (DOC) [file pone.0105110.s001.doc]

Table S1: Period prevalence rates for boys stratified by age for the year 2008. SABA: Short-acting beta-2-agonist, CGA: Cromoglicic acid, LABA: Long-acting beta-2-agonist, ICS: Inhaled corticosteroid, SAMA: Short-acting muscarinic antagonist, LAMA: Long-acting muscarinic antagonist, B2A: Beta-2-agonist.

| **Compound class** | **Compound** | **0** | **1** | **2** | **3** | **4** | **5** | **6** | **7** | **8** | **9** | **10** | **11** | **12** | **13** | **14** | **15** | **16** | **17** | **18** |
| --- | --- | --- | --- | --- | --- | --- | --- | --- | --- | --- | --- | --- | --- | --- | --- | --- | --- | --- | --- | --- |
| **Inhaled SABA** | **Salbutamol** | 1148.8 | 1118.0 | 916.6 | 1097.8 | 991.0 | 873.9 | 754.7 | 636.7 | 610.9 | 571.6 | 558.7 | 553.5 | 473.8 | 395.6 | 336.2 | 314.6 | 291.2 | 292.9 | 201.4 |
|  | **Fenoterol** | 0.0 | 0.4 | 0.4 | 1.3 | 1.9 | 1.6 | 2.2 | 3.5 | 4.9 | 7.1 | 8.4 | 7.6 | 8.9 | 7.3 | 6.6 | 10.9 | 10.2 | 12.6 | 10.8 |
|  | **Terbutaline** | 0.0 | 0.0 | 0.0 | 0.0 | 0.0 | 0.0 | 0.6 | 0.6 | 0.2 | 0.7 | 0.7 | 2.1 | 0.9 | 1.4 | 0.3 | 1.2 | 1.8 | 2.6 | 1.5 |
| **Inhaled SABA combination** | **Fenoterol/Ipratropium (fixed combination)** | 35.3 | 31.9 | 25.4 | 26.6 | 23.8 | 22.8 | 18.0 | 14.0 | 11.6 | 16.3 | 15.5 | 17.9 | 13.8 | 12.8 | 10.1 | 13.9 | 11.6 | 13.9 | 12.2 |
|  | **Reproterol/CGA (fixed combination)** | 0.6 | 0.9 | 2.6 | 2.3 | 6.6 | 11.6 | 15.2 | 17.7 | 25.3 | 32.4 | 40.9 | 46.0 | 49.5 | 52.5 | 54.8 | 59.1 | 61.4 | 68.5 | 53.6 |
| **Inhaled LABA** | **Salmeterol** | 0.0 | 0.2 | 0.2 | 0.8 | 2.3 | 2.4 | 2.6 | 3.5 | 2.2 | 2.2 | 2.5 | 3.3 | 2.3 | 2.8 | 1.7 | 2.0 | 1.8 | 1.1 | 0.6 |
|  | **Formoterol** | 0.4 | 0.0 | 0.0 | 0.4 | 2.3 | 5.3 | 10.8 | 15.7 | 23.4 | 24.0 | 25.7 | 29.0 | 28.4 | 25.1 | 22.9 | 24.0 | 20.3 | 23.9 | 20.5 |
| **Inhaled LABA/ICS** | **Salmeterol/Fluticasone (fixed combination)** | 2.4 | 8.5 | 12.7 | 29.4 | 62.6 | 86.0 | 104.1 | 111.4 | 126.1 | 135.1 | 141.9 | 146.2 | 136.4 | 117.2 | 105.9 | 94.6 | 81.7 | 76.7 | 55.7 |
|  | **Formoterol/Beclomethasone (fixed combination)** | 0.0 | 0.2 | 0.4 | 0.2 | 0.6 | 0.6 | 1.0 | 0.6 | 1.7 | 2.4 | 4.6 | 4.7 | 8.5 | 11.0 | 15.1 | 18.4 | 27.2 | 25.0 | 27.2 |
|  | **Formoterol/Budesonide (fixed combination)** | 0.2 | 0.0 | 0.2 | 1.5 | 3.5 | 15.0 | 23.4 | 36.9 | 56.2 | 64.8 | 79.9 | 97.8 | 90.0 | 82.2 | 68.7 | 69.6 | 68.3 | 74.1 | 55.4 |
| **Inhaled SAMA** | **Ipratropium** | 375.7 | 302.0 | 223.0 | 236.0 | 206.5 | 164.7 | 130.1 | 89.6 | 82.4 | 65.4 | 46.0 | 39.8 | 30.9 | 19.7 | 12.5 | 12.8 | 7.4 | 5.8 | 3.1 |
| **Inhaled LAMA** | **Tiotropium** | 0.0 | 0.0 | 0.0 | 0.0 | 0.2 | 0.0 | 0.0 | 0.2 | 0.0 | 0.2 | 0.4 | 0.0 | 0.3 | 0.4 | 0.7 | 0.2 | 1.0 | 1.1 | 0.3 |
| **ICS** | **Budesonide** | 207.3 | 231.7 | 205.8 | 261.1 | 255.5 | 238.3 | 210.8 | 186.6 | 184.5 | 159.5 | 162.6 | 159.8 | 136.1 | 112.1 | 92.0 | 98.2 | 87.5 | 106.8 | 79.1 |
|  | **Beclomethasone** | 133.4 | 159.5 | 140.1 | 180.2 | 151.5 | 140.3 | 129.7 | 109.8 | 105.5 | 101.4 | 86.4 | 90.3 | 78.3 | 54.4 | 50.4 | 38.9 | 39.6 | 40.3 | 29.2 |
|  | **Fluticasone** | 38.3 | 75.0 | 67.3 | 95.0 | 123.4 | 128.5 | 115.5 | 88.0 | 81.9 | 67.6 | 66.9 | 58.4 | 45.5 | 36.5 | 29.4 | 23.5 | 12.7 | 12.5 | 10.2 |
|  | **Ciclesonide** | 0.2 | 0.2 | 0.0 | 0.0 | 0.0 | 0.6 | 0.2 | 0.4 | 0.4 | 0.5 | 0.4 | 0.9 | 1.7 | 1.6 | 2.8 | 2.3 | 1.7 | 2.7 | 1.2 |
| **Oral B2A** | **Salbutamol** | 375.4 | 421.5 | 297.0 | 275.6 | 198.2 | 130.9 | 75.1 | 54.4 | 42.5 | 36.1 | 23.7 | 17.7 | 14.7 | 9.1 | 6.6 | 2.8 | 1.5 | 1.6 | 0.8 |
|  | **Terbutaline** | 118.9 | 150.6 | 102.3 | 110.1 | 82.5 | 60.8 | 42.8 | 29.7 | 30.2 | 21.6 | 13.6 | 10.2 | 6.5 | 3.4 | 2.1 | 2.7 | 2.0 | 1.4 | 0.6 |
|  | **Tulobuterol** | 4.7 | 15.8 | 17.8 | 17.8 | 16.9 | 11.2 | 10.2 | 6.2 | 6.2 | 4.4 | 4.6 | 4.0 | 2.4 | 0.7 | 0.2 | 0.7 | 0.2 | 0.2 | 0.0 |
|  | **Clenbuterol** | 0.6 | 1.1 | 0.6 | 0.4 | 1.0 | 1.0 | 0.4 | 0.2 | 0.6 | 0.2 | 1.4 | 0.7 | 0.5 | 0.7 | 0.0 | 0.2 | 0.2 | 0.3 | 0.5 |
| **Oral B2A combination** | **Clenbuterol/Ambroxol (fixed combination)** | 1091.0 | 1331.6 | 1000.9 | 1074.7 | 872.7 | 650.0 | 491.4 | 379.0 | 336.1 | 282.2 | 246.4 | 215.0 | 158.4 | 125.1 | 89.0 | 79.3 | 75.9 | 69.0 | 53.6 |
| **Others** | **Theophylline** | 4.3 | 2.3 | 3.7 | 2.9 | 3.3 | 2.6 | 4.0 | 4.1 | 2.2 | 3.7 | 4.8 | 3.5 | 3.8 | 6.2 | 7.5 | 7.1 | 5.6 | 6.1 | 9.1 |
|  | **Montelukast** | 121.0 | 217.0 | 191.7 | 237.8 | 229.7 | 208.8 | 171.6 | 139.3 | 128.5 | 117.0 | 114.7 | 106.1 | 89.0 | 68.3 | 50.1 | 43.9 | 33.7 | 29.1 | 20.1 |
|  | **Cromoglicic acid** | 22.1 | 31.1 | 35.5 | 43.0 | 52.3 | 49.6 | 41.2 | 32.7 | 35.0 | 27.6 | 22.3 | 29.4 | 11.2 | 4.3 | 2.4 | 2.3 | 1.5 | 1.1 | 0.6 |
