# Supplementary material for: Utilisation and Off-Label Prescriptions of Respiratory Drugs in Children
Source: PLoS One. 2014 Sep 2;9(9):e105110. doi: 10.1371/journal.pone.0105110 (PMC4152124; doi:10.1371/journal.pone.0105110)
Supplement: Table S2 — Period prevalence rates for girls stratified by age for the year 2008. SABA: Short-acting beta-2-agonist, CGA: Cromoglicic Acid, LABA: Long-acting beta-2-agonist, ICS: Inhaled corticosteroid, SAMA: Short-acting muscarinic antagonist, LAMA: Long-acting muscarinic antagonist, B2A: Beta-2-agonist. (DOC) [file pone.0105110.s002.doc]

Table S2: Period prevalence rates for girls stratified by age for the year 2008. SABA: Short-acting beta-2-agonist, CGA: Cromoglicic acid, LABA: Long-acting beta-2-agonist, ICS: Inhaled corticosteroid, SAMA: Short-acting muscarinic antagonist, LAMA: Long-acting muscarinic antagonist, B2A: Beta-2-agonist.

| **Compound class** | **Compound** | **0** | **1** | **2** | **3** | **4** | **5** | **6** | **7** | **8** | **9** | **10** | **11** | **12** | **13** | **14** | **15** | **16** | **17** | **18** |
| --- | --- | --- | --- | --- | --- | --- | --- | --- | --- | --- | --- | --- | --- | --- | --- | --- | --- | --- | --- | --- |
| **Inhaled SABA** | **Salbutamol** | 790.1 | 760.6 | 693.9 | 814.1 | 731.0 | 617.0 | 488.4 | 419.1 | 380.1 | 364.3 | 352.0 | 324.9 | 294.2 | 269.1 | 258.0 | 285.1 | 298.6 | 316.2 | 254.4 |
|  | **Fenoterol** | 0.0 | 0.0 | 0.5 | 0.9 | 1.1 | 0.9 | 1.9 | 2.3 | 2.7 | 2.5 | 4.7 | 4.2 | 4.2 | 6.4 | 5.9 | 10.2 | 9.3 | 12.9 | 13.0 |
|  | **Terbutaline** | 0.0 | 0.0 | 0.0 | 0.0 | 0.2 | 0.0 | 0.2 | 0.4 | 0.2 | 0.8 | 0.2 | 1.1 | 0.7 | 0.8 | 0.6 | 1.2 | 1.4 | 1.5 | 0.6 |
| **Inhaled SABA combination** | **Fenoterol/Ipratropium (fixed combination)** | 28.8 | 17.7 | 14.1 | 20.8 | 20.6 | 12.3 | 12.2 | 10.3 | 6.5 | 10.3 | 10.4 | 9.2 | 5.9 | 8.6 | 12.4 | 10.5 | 15.4 | 16.7 | 12.5 |
|  | **Reproterol/CGA (fixed combination)** | 0.5 | 0.4 | 0.9 | 2.9 | 3.0 | 5.5 | 7.8 | 12.2 | 16.0 | 23.6 | 20.6 | 33.9 | 27.0 | 36.2 | 38.0 | 45.0 | 53.1 | 62.5 | 49.2 |
| **Inhaled LABA** | **Salmeterol** | 0.0 | 0.2 | 0.0 | 0.2 | 0.9 | 1.5 | 1.3 | 1.7 | 1.4 | 1.4 | 1.9 | 1.5 | 1.5 | 0.9 | 0.9 | 1.4 | 1.5 | 1.4 | 1.3 |
|  | **Formoterol** | 0.0 | 0.0 | 0.2 | 0.7 | 0.6 | 5.1 | 7.1 | 10.3 | 11.7 | 14.4 | 16.0 | 17.2 | 15.9 | 19.5 | 17.0 | 18.6 | 19.9 | 21.5 | 21.7 |
| **Inhaled LABA/ICS** | **Salmeterol/Fluticasone (fixed combination)** | 0.9 | 3.5 | 6.6 | 19.5 | 41.6 | 52.5 | 58.6 | 72.8 | 64.1 | 80.0 | 81.3 | 81.5 | 72.4 | 64.0 | 58.8 | 65.1 | 65.0 | 71.0 | 54.5 |
|  | **Formoterol/Beclomethasone (fixed combination)** | 0.2 | 0.0 | 0.2 | 0.2 | 0.2 | 0.2 | 0.6 | 0.2 | 1.4 | 1.6 | 3.0 | 3.3 | 7.9 | 12.2 | 10.3 | 21.7 | 26.7 | 35.9 | 38.3 |
|  | **Formoterol/Budesonide (fixed combination)** | 0.2 | 0.0 | 0.7 | 0.4 | 1.7 | 5.3 | 14.9 | 22.5 | 31.1 | 36.3 | 42.1 | 51.3 | 52.1 | 47.3 | 50.3 | 56.9 | 63.4 | 77.0 | 75.0 |
| **Inhaled SAMA** | **Ipratropium** | 227.5 | 200.3 | 150.8 | 167.0 | 131.4 | 105.0 | 72.9 | 61.4 | 46.9 | 43.0 | 31.7 | 23.6 | 16.4 | 13.5 | 7.9 | 6.3 | 5.8 | 6.3 | 4.2 |
| **Inhaled LAMA** | **Tiotropium** | 0.0 | 0.0 | 0.0 | 0.0 | 0.0 | 0.0 | 0.2 | 0.2 | 0.2 | 0.0 | 0.2 | 0.4 | 0.0 | 0.8 | 0.4 | 0.9 | 1.2 | 1.7 | 1.0 |
| **ICS** | **Budesonide** | 119.1 | 142.4 | 142.6 | 174.7 | 175.4 | 153.9 | 124.1 | 118.5 | 98.5 | 109.1 | 92.4 | 86.3 | 82.2 | 70.4 | 76.2 | 87.7 | 108.5 | 115.1 | 107.7 |
|  | **Beclomethasone** | 68.1 | 95.4 | 88.9 | 131.1 | 110.5 | 95.5 | 86.9 | 74.9 | 65.5 | 70.1 | 61.1 | 54.8 | 52.3 | 42.4 | 45.0 | 43.9 | 55.0 | 52.6 | 47.7 |
|  | **Fluticasone** | 14.9 | 32.8 | 46.9 | 68.4 | 85.4 | 84.8 | 71.2 | 52.7 | 45.2 | 46.5 | 42.4 | 33.7 | 26.2 | 17.1 | 19.4 | 12.6 | 13.0 | 7.7 | 6.7 |
|  | **Ciclesonide** | 0.0 | 0.7 | 0.2 | 0.2 | 0.0 | 0.0 | 0.6 | 0.0 | 0.4 | 0.2 | 0.6 | 0.9 | 1.5 | 2.3 | 3.0 | 3.0 | 3.3 | 5.1 | 2.9 |
| **Oral B2A** | **Salbutamol** | 257.6 | 322.2 | 241.0 | 234.1 | 163.5 | 109.9 | 73.9 | 47.6 | 34.4 | 27.7 | 16.4 | 13.4 | 7.8 | 6.0 | 4.2 | 4.2 | 1.2 | 2.7 | 1.8 |
|  | **Terbutaline** | 69.0 | 115.8 | 93.2 | 87.0 | 65.5 | 47.6 | 39.7 | 31.4 | 21.5 | 16.8 | 8.9 | 7.5 | 2.6 | 2.8 | 2.2 | 2.8 | 0.9 | 1.2 | 0.8 |
|  | **Tulobuterol** | 3.4 | 11.8 | 13.2 | 19.9 | 10.5 | 11.3 | 5.7 | 7.2 | 4.5 | 2.1 | 2.8 | 1.5 | 1.1 | 0.9 | 0.7 | 0.5 | 0.3 | 0.0 | 0.2 |
|  | **Clenbuterol** | 0.2 | 0.0 | 0.5 | 0.0 | 0.6 | 0.2 | 0.6 | 0.0 | 1.0 | 0.2 | 0.0 | 0.0 | 0.4 | 0.4 | 0.2 | 0.4 | 0.9 | 1.2 | 2.1 |
| **Oral B2A combination** | **Clenbuterol/Ambroxol (fixed combination)** | 762.0 | 994.2 | 875.3 | 899.5 | 718.4 | 542.8 | 397.5 | 292.9 | 255.0 | 221.4 | 178.1 | 144.5 | 99.7 | 78.3 | 69.5 | 70.7 | 75.2 | 79.5 | 62.3 |
| **Others** | **Theophylline** | 2.3 | 2.0 | 2.0 | 2.9 | 3.0 | 2.6 | 2.1 | 2.9 | 2.5 | 2.3 | 2.6 | 4.0 | 3.1 | 3.8 | 5.0 | 6.3 | 8.7 | 10.6 | 11.8 |
|  | **Montelukast** | 68.4 | 116.9 | 130.6 | 170.3 | 162.0 | 139.3 | 117.8 | 91.0 | 80.6 | 80.8 | 66.0 | 59.2 | 44.5 | 42.0 | 36.1 | 33.8 | 41.1 | 41.9 | 26.8 |
|  | **Cromoglicic acid** | 16.5 | 24.0 | 28.8 | 45.4 | 41.4 | 35.5 | 31.9 | 23.6 | 19.6 | 20.5 | 17.5 | 17.2 | 5.5 | 2.3 | 1.5 | 2.6 | 1.2 | 1.5 | 0.3 |
