# Supplementary material for: Utilisation and Off-Label Prescriptions of Respiratory Drugs in Children
Source: PLoS One. 2014 Sep 2;9(9):e105110. doi: 10.1371/journal.pone.0105110 (PMC4152124; doi:10.1371/journal.pone.0105110)
Supplement: Table S3 — Number and proportion of off-label indications (year 2008, multiple counting of off-label indications per prescription). SABA: Short-acting beta-2-agonist, CGA: Cromoglicic Acid, LABA: Long-acting beta-2-agonist, ICS: Inhaled corticosteroid, SAMA: Short-acting muscarinic antagonist, LAMA: Long-acting muscarinic antagonist, B2A: Beta-2-agonist, nec: not elsewhere classified, n.a.: not applicable (i.e. labeled diagnoses). (DOC) [file pone.0105110.s003.doc]

Table S3: Number and proportion of off-label indications (year 2008, multiple counting of off-label indications per prescription). SABA: Short-acting beta-2-agonist, CGA: Cromoglicic acid, LABA: Long-acting beta-2-agonist, ICS: Inhaled corticosteroid, SAMA: Short-acting muscarinic antagonist, LAMA: Long-acting muscarinic antagonist, B2A: Beta-2-agonist, nec: not elsewhere classified, n.a.: not applicable (i.e. labeled diagnoses).

| **Compound class** | **Compound** | **All prescriptions (n)** | **Off-label due to indication or age&indication**  **(n, % of all prescriptions)** | **Off-label indications (n, % of all off-label prescriptions due to ‘indication’ and ‘age&indication’)** | | | | | | | | | | | |
| --- | --- | --- | --- | --- | --- | --- | --- | --- | --- | --- | --- | --- | --- | --- | --- |
|  |  |  |  | **Acute upper respiratory tract infections** | **Other diseases of upper respiratory tract** | **Influenza and pneumonia** | **Emphysema** | **Acute bronch-itis** | **Acute bronch-iolitis / Unspecified acute lower respiratory tract infection** | **Bron-chitis nec** | **Chronic bronchitis** | **Other respiratory diseases** | **COPD** | **Asthma** | **Missing indication** |
| **Inhaled SABA** | **Salbutamol** | 159,655 | 67,084 (42.0%) | 23,827 (35.5%) | 13,267 (19.8%) | 4,056 (6.0%) | n.a. | 29,989 (44.7%) | 2,309 (3.4%) | 11,891 (17.7%) | 2,407 (3.6%) | 6,470 (9.6%) | n.a. | n.a | 10,489 (15.6%) |
|  | **Fenoterol** | 1,452 | 377 (26.0%) | 56 (14.9%) | 86 (22.8%) | 6 (1.6%) | n.a. | 38 (10.1%) | 3 (0.8%) | 57 (15.1%) | 18 (4.8%) | 13 (3.4%) | n.a. | n.a | 164 (43.5%) |
|  | **Terbutaline** | 184 | 34 (18.5%) | 10 (29.4%) | 10 (29.4%) | 0 | n.a. | 5 (14.7%) | 0 | 6 (17.6%) | 1 (2.9%) | 1 (2.9%) | n.a. | n.a | 10 (29.4%) |
| **Inhaled SABA combination** | **Fenoterol/Ipratropium (fixed combination)** | 3,998 | 1,722 (43.1%) | 551 (32.0%) | 291 (16.9%) | 104 (6.0%) | 0 | 702 (40.8%) | 75 (4.4%) | 343 (19.9%) | 56 (3.3%) | 113 (6.6%) | n.a. | n.a | 344 (20.0%) |
|  | **Reproterol/CGA (fixed combination)** | 8,729 | 2,538 (29.1%) | 402 (15.8%) | 999 (39.4%) | 28 (1.1%) | 0 | 253 (10.0%) | 17 (0.7%) | 259 (10.2%) | 72 (2.8%) | 99 (3.9%) | 214 (8.4%) | n.a | 815 (32.1%) |
| **Inhaled SABA (incl. combination) Total** | | **174,018** | **71,755 (41.2%)** | **24,846 (34.6%)** | **14,653 (20.4%)** | **4,194 (5.8%)** | **0 / n.a. (0% / n.a.)** | **30,987 (43.2%)** | **2,404 (3.4%)** | **12,556 (17.5%)** | **2,554 (3.6%)** | **6,696 (9.3%)** | **214 / n.a. (0.3% /n.a.)** | **n.a.** | **11,822 (16.5%)** |
| **Inhaled LABA** | **Salmeterol** | 522 | 77 (14.8%) | 13 (16.9%) | 16 (20.8%) | 3 (3.9%) | 0 | 12 (15.6%) | 0 | 9 (11.7%) | 1 (1.3%) | 5 (6.5%) | n.a. | n.a | 31 (40.3%) |
|  | **Formoterol** | 4,931 | 751 (15.2%) | 133 (17.7%) | 160 (21.3%) | 12 (1.6%) | 1 (0.1%) | 121 (16.1%) | 10 (1.3%) | 108 (14.4%) | 21 (2.8%) | 39 (5.2%) | n.a. | n.a | 285 (37.9%) |
| **Inhaled LABA/ICS** | **Salmeterol/Fluticasone (fixed combination)** | 27,600 | 3,910 (14.2%) | 679 (17.4%) | 1,066 (27.3%) | 101 (2.6%) | 5 (0.1%) | 751 (19.2%) | 51 (1.3%) | 592 (15.1%) | 155 (4.0%) | 201 (5.1%) | n.a. | n.a | 1,343 (34.3%) |
|  | **Formoterol/Beclomethasone (fixed combination)** | 2,515 | 837 (33.3%) | 171 (20.4%) | 153 (18.3%) | 17 (2.0%) | 0 | 130 (15.5%) | 16 (1.9%) | 149 (17.8%) | 58 (6.9%) | 36 (4.3%) | 107 (12.8%) | n.a | 240 (28.7%) |
|  | **Formoterol/Budesonide (fixed combination)** | 13,833 | 2,471 (17.9%) | 456 (18.5%) | 621 (25.1%) | 48 (1.9%) | 1 (0.0%) | 392 (15.9%) | 33 (1.3%) | 428 (17.3%) | 109 (4.4%) | 129 (5.2%) | n.a. | n.a | 875 (35.4%) |
| **Inhaled LABA (incl. combination) Total** | | **49,401** | **8,046 (16.3%)** | **1,452 (18.0%)** | **2,016 (25.1%)** | **181 (2.2%)** | **7 (0.0%)** | **1,406 (17.5%)** | **110 (1.4%)** | **1,286 (16.0%)** | **344 (4.3%)** | **410 (5.1%)** | **107 / n.a. (1.3% / n.a.)** | **n.a.** | **2,774 (34.5%)** |
| **Inhaled SAMA** | **Ipratropium** | 21,822 | 10,910 (50.0%) | 4,124 (37.8%) | 1,804 (16.5%) | 871 (8.0%) | 1 (0.0%) | 5,779 (53.0%) | 393 (3.6%) | 2,033 (18.6%) | 421 (3.9%) | 1,266 (11.6%) | n.a. | n.a | 1,268 (11.6%) |
| **Inhaled LAMA** | **Tiotropium** | 97 | 87 (89.7%) | 14 (16.1%) | 18 (20.7%) | 3 (3.4%) | 0 | 8 (9.2%) | 2 (2.3%) | 6 (6.9%) | 2 (2.3%) | 1 (1.1%) | n.a. | 61 (70.1%) | 9 (10.3%) |
| **Muscarinic antagonists (SAMA & LAMA) total** | | **21,919** | **10,997 (50.2%)** | **4,138 (37.6%)** | **1,822 (16.6%)** | **874 (7.9%)** | **1 (0.0%)** | **5,787 (52.6%)** | **395 (3.6%)** | **2,039 (18.5%)** | **423 (3.8%)** | **1,267 (11.5%)** | **n.a.** | **61 / n.a. (0.6% / n.a.)** | **1,277 (11.6%)** |
| **Inhaled bronchodilative drugs (SABA, LABA, SAMA, LAMA [incl. combination]) Total** | | **245,338** | **90,798 (37.0%)** | **30,436 (33.5%)** | **18,491 (20.4%)** | **5,249 (5.8%)** | **8 (0.0%)** | **38,180 (42.0%)** | **2,909 (3.2%)** | **15,881 (17.5%)** | **3,321 (3.7%)** | **8,373 (9.2%)** | **321 / n.a. (0.4%, n.a.)** | **61 / n.a. (0.1%, n.a.)** | **15,873 (17.5%)** |
| **ICS** | **Budesonide** | 42,067 | 3,166 (7.5%) | n.a. | n.a. | n.a. | n.a. | n.a. | n.a. | n.a. | n.a. | n.a. | n.a. | n.a | 3,166 (100.0%) |
|  | **Beclomethasone** | 24,185 | 1,922 (7.9%) | n.a. | n.a. | n.a. | n.a. | n.a. | n.a. | n.a. | n.a. | n.a. | n.a. | n.a | 1,922 (100.0%) |
|  | **Fluticasone** | 17,097 | 3,443 (20.1%) | 977 (28.4%) | 847 (24.6%) | 132 (3.8%) | 0 | 1,096 (31.8%) | 57 (1.7%) | 502 (14.6%) | 174 (5.1%) | 260 (7.6%) | n.a. | n.a | 854 (24.8%) |
|  | **Ciclesonide** | 326 | 75 (23.0%) | 11 (14.7%) | 20 (26.7%) | 2 (2.7%) | 0 | 14 (18.7%) | 0 | 12 (16.0%) | 1 (1.3%) | 2 (2.7%) | 5 (6.7%) | n.a | 29 (38.7%) |
| **ICS Total** | | **83,675** | **8,606 (10.3%)** | **988 / n.a. (11.5% / n.a.)** | **867 / n.a. (10.1% / n.a.)** | **134 / n.a. (1.6% / n.a.)** | **0 / n.a. (0% / n.a.)** | **1,110 / n.a. (12.9% / n.a.)** | **57 / n.a. (0.7% / n.a.)** | **514 / n.a. (6.0% / n.a.)** | **175 / n.a. (2.0% / n.a.)** | **262 / n.a. (3.0% / n.a.)** | **5 / n.a. (0.1% / n.a.)** | **n.a.** | **5,971 (69.4%)** |
| **Oral B2A** | **Salbutamol** | 19,475 | 5,544 (28.5%) | 2,431 (43.8%) | 1,001 (18.1%) | 346 (6.2%) | n.a. | n.a. | 499 (9.0%) | 2,020 (36.4%) | 234 (4.2%) | 793 (14.3%) | n.a. | n.a | 870 (15.7%) |
|  | **Terbutaline** | 6,940 | 2,012 (29.0%) | 880 (43.7%) | 348 (17.3%) | 131 (6.5%) | n.a. | n.a. | 132 (6.6%) | 773 (38.4%) | 52 (2.6%) | 192 (9.5%) | n.a. | n.a | 302 (15.0%) |
|  | **Tulobuterol** | 1,201 | 298 (24.8%) | 110 (36.9%) | 63 (21.1%) | 23 (7.7%) | n.a. | n.a. | 5 (1.7%) | 125 (41.9%) | 25 (8.4%) | 22 (7.4%) | n.a. | n.a | 49 (16.4%) |
|  | **Clenbuterol** | 113 | 67 (59.3%) | 11 (16.4%) | 3 (4.5%) | 1 (1.5%) | n.a. | n.a. | 4 (6.0%) | 10 (14.9%) | n.a. | 6 (9.0%) | n.a. | n.a | 40 (59.7%) |
| **Oral B2A combination** | **Clenbuterol/Ambroxol (fixed combination)** | 91,385 | 18,897 (20.7%) | 9,131 (48.3%) | 2,767 (14.6%) | 1,501 (7.9%) | n.a. | n.a. | n.a. | n.a. | n.a. | 2,228 (11.8%) | n.a. | n.a | 6,035 (31.9%) |
| **Oral B2A (incl. combination) Total** | | **119,114** | **26,818 (22.5%)** | **12,563 (46.8%)** | **4,182 (15.6%)** | **2,002 (7.5%)** | **n.a.** | **n.a.** | **640 / n.a. (2.4% / n.a.)** | **2,928 / n.a. (10.9% / n.a.)** | **311 / n.a. (1.2% / n.a.)** | **3,241 (12.1%)** | **n.a.** | **n.a.** | **7,296 (27.2%)** |
| **Others** | **Theophylline** | 1,184 | 444 (37.5%) | 147 (33.1%) | 83 (18.7%) | 17 (3.8%) | 1 (0.2%) | 125 (28.2%) | 9 (2.0%) | 149 (33.6%) | 26 (5.9%) | 38 (8.6%) | n.a. | n.a | 90 (20.3%) |
|  | **Montelukast** | 33,501 | 12,522 (37.4%) | 3,850 (30.7%) | 3,014 (24.1%) | 503 (4.0%) | 1 (0.0%) | 3,868 (30.9%) | 281 (2.2%) | 1,879 (15.0%) | 665 (5.3%) | 1,051 (8.4%) | 1,700 (13.6%) | n.a | 2,539 (20.3%) |
|  | **Cromoglicic acid** | 5,087 | 3,137 (61.7%) | 1,151 (36.7%) | 789 (25.2%) | 133 (4.2%) | 0 | 1,101 (35.1%) | 106 (3.4%) | 544 (17.3%) | 114 (3.6%) | 179 (5.7%) | 381 (12.1%) | n.a | 572 (18.2%) |
| **Others Total** | | **39,772** | **16,103 (40.5%)** | **5,148 (32.0%)** | **3,886 (24.1%)** | **653 (4.1%)** | **2 (0.0%)** | **5,094 (31.6%)** | **396 (2.5%)** | **2,572 (16.0%)** | **805 (5.0%)** | **1,268 (7.9%)** | **2,081 (12.9%)** | **n.a.** | **3,201 (19.9%)** |
| **All drugs Total** | | **487,899** | **142,325 (29.2%)** | **49,135 / n.a. (34.5% / n.a.)** | **27,426 / n.a. (19.3% / n.a.)** | **8,038 / n.a. (5.6% / n.a.)** | **10 / n.a. (0.0% / n.a.)** | **44,384 / n.a. (31.2% / n.a.)** | **4,002 / n.a. (2.8% / n.a.)** | **21,895 / n.a. (15.4% / n.a.)** | **4,612 / n.a. (3.2% / n.a.)** | **13,144 / n.a. (9.2% / n.a.)** | **2,407 / n.a. (1.7% / n.a.)** | **61 / n.a. (0.0% / n.a.)** | **32,341 (22.7%)** |
