# Supplementary material for: Utilisation and Off-Label Prescriptions of Respiratory Drugs in Children
Source: PLoS One. 2014 Sep 2;9(9):e105110. doi: 10.1371/journal.pone.0105110 (PMC4152124; doi:10.1371/journal.pone.0105110)
Supplement: Table S4 — Number and proportion of off-label prescriptions stratified by off-label type, gender, and age group (year 2008). SABA: Short-acting beta-2-agonist, CGA: Cromoglicic Acid, LABA: Long-acting beta-2-agonist, ICS: Inhaled corticosteroid, SAMA: Short-acting muscarinic antagonist, LAMA: Long-acting muscarinic antagonist, B2A: Beta-2-agonist. (DOC) [file pone.0105110.s004.doc]

Table S4: Number and proportion of off-label prescriptions stratified by off-label type, gender, and age group (year 2008). SABA: Short-acting beta-2-agonist, CGA: Cromoglicic acid, LABA: Long-acting beta-2-agonist, ICS: Inhaled corticosteroid, SAMA: Short-acting muscarinic antagonist, LAMA: Long-acting muscarinic antagonist, B2A: Beta-2-agonist.

| **Compound class** | **Compound** | **Age group** | **Male** | | | | | **Female** | | | | |
| --- | --- | --- | --- | --- | --- | --- | --- | --- | --- | --- | --- | --- |
|  |  |  | **All prescriptions (n)** | **Off-label overall**  **(n,%)** | **Off-label due to age only (n)** | **Off-label due to indication only (n)** | **Off-label due to age and indication (n)** | **All prescriptions (n)** | **Off-label overall**  **(n,%)** | **Off-label due to age only (n)** | **Off-label due to indication only (n)** | **Off-label due to age and indication (n)** |
| **Inhaled SABA** | **Salbutamol** | 0-1 | 15,320 | 9,632 (62.9%) | 0 | 9,632 | 0 | 9,369 | 6,306 (67.3%) | 0 | 6,306 | 0 |
|  |  | 2-5 | 29,961 | 15,090 (50.4%) | 0 | 15,090 | 0 | 20,317 | 11,161 (54.9%) | 0 | 11,161 | 0 |
|  |  | 6-11 | 30,262 | 9,251 (30.6%) | 0 | 9,251 | 0 | 17,770 | 6,110 (34.4%) | 0 | 6,110 | 0 |
|  |  | 12-14 | 10,417 | 2,299 (22.1%) | 0 | 2,299 | 0 | 6,473 | 1,892 (29.2%) | 0 | 1,892 | 0 |
|  |  | 15-18 | 10,145 | 2,349 (23.2%) | 0 | 2,349 | 0 | 9,621 | 2,994 (31.1%) | 0 | 2,994 | 0 |
|  | **Fenoterol** | 0-1 | 2 | 2 (100.0%) | 0 | 0 | 2 | 0 | 0 | 0 | 0 | 0 |
|  |  | 2-5 | 28 | 16 (57.1%) | 4 | 8 | 4 | 16 | 8 (50.0%) | 2 | 2 | 4 |
|  |  | 6-11 | 230 | 62 (27.0%) | 0 | 62 | 0 | 115 | 32 (27.8%) | 0 | 32 | 0 |
|  |  | 12-14 | 166 | 41 (24.7%) | 0 | 41 | 0 | 116 | 35 (30.2%) | 0 | 35 | 0 |
|  |  | 15-18 | 411 | 81 (19.7%) | 0 | 81 | 0 | 368 | 106 (28.8%) | 0 | 106 | 0 |
|  | **Terbutaline** | 0-1 | 0 | 0 | 0 | 0 | 0 | 0 | 0 | 0 | 0 | 0 |
|  |  | 2-5 | 0 | 0 | 0 | 0 | 0 | 1 | 1 (100.0%) | 0 | 0 | 1 |
|  |  | 6-11 | 31 | 7 (22.6%) | 0 | 7 | 0 | 15 | 4 (26.7%) | 0 | 4 | 0 |
|  |  | 12-14 | 24 | 3 (12.5%) | 0 | 3 | 0 | 16 | 6 (37.5%) | 0 | 6 | 0 |
|  |  | 15-18 | 63 | 6 (9.5%) | 0 | 6 | 0 | 34 | 7 (20.6%) | 0 | 7 | 0 |
| **Inhaled SABA combination** | **Fenoterol/ Ipratropium (fixed combination)** | 0-1 | 360 | 228 (63.3%) | 0 | 228 | 0 | 242 | 173 (71.5%) | 0 | 173 | 0 |
|  |  | 2-5 | 630 | 332 (52.7%) | 0 | 332 | 0 | 379 | 225 (59.4%) | 0 | 225 | 0 |
|  |  | 6-11 | 644 | 234 (36.3%) | 0 | 234 | 0 | 365 | 152 (41.6%) | 0 | 152 | 0 |
|  |  | 12-14 | 296 | 74 (25.0%) | 0 | 74 | 0 | 188 | 55 (29.3%) | 0 | 55 | 0 |
|  |  | 15-18 | 444 | 108 (24.3%) | 0 | 108 | 0 | 450 | 141 (31.3%) | 0 | 141 | 0 |
|  | **Reproterol/CGA (fixed combination)** | 0-1 | 7 | 3 (42.9%) | 0 | 3 | 0 | 4 | 3 (75.0%) | 0 | 3 | 0 |
|  |  | 2-5 | 137 | 72 (52.6%) | 0 | 72 | 0 | 63 | 37 (58.7%) | 0 | 37 | 0 |
|  |  | 6-11 | 1,297 | 390 (30.1%) | 0 | 390 | 0 | 811 | 275 (33.9%) | 0 | 275 | 0 |
|  |  | 12-14 | 1,321 | 349 (26.4%) | 0 | 349 | 0 | 785 | 240 (30.6%) | 0 | 240 | 0 |
|  |  | 15-18 | 2,361 | 584 (24.7%) | 0 | 584 | 0 | 1,943 | 585 (30.1%) | 0 | 585 | 0 |
| **Inhaled LABA** | **Salmeterol** | 0-1 | 1 | 1 (100.0%) | 1 | 0 | 0 | 3 | 3 (100.0%) | 3 | 0 | 0 |
|  |  | 2-5 | 40 | 16 (40.0%) | 4 | 10 | 2 | 15 | 2 (13.3%) | 1 | 1 | 0 |
|  |  | 6-11 | 161 | 24 (14.9%) | 0 | 24 | 0 | 67 | 13 (19.4%) | 0 | 13 | 0 |
|  |  | 12-14 | 75 | 14 (18.7%) | 0 | 14 | 0 | 36 | 2 (5.6%) | 0 | 2 | 0 |
|  |  | 15-18 | 72 | 2 (2.8%) | 0 | 2 | 0 | 52 | 9 (17.3%) | 0 | 9 | 0 |
|  | **Formoterol** | 0-1 | 2 | 2 (100.0%) | 0 | 0 | 2 | 0 | 0 | 0 | 0 | 0 |
|  |  | 2-5 | 65 | 65 (100.0%) | 54 | 0 | 11 | 42 | 42 (100.0%) | 34 | 0 | 8 |
|  |  | 6-11 | 1,245 | 143 (11.5%) | 0 | 143 | 0 | 711 | 93 (13.1%) | 0 | 93 | 0 |
|  |  | 12-14 | 727 | 103 (14.2%) | 0 | 103 | 0 | 468 | 79 (16.9%) | 0 | 79 | 0 |
|  |  | 15-18 | 927 | 152 (16.4%) | 0 | 152 | 0 | 744 | 160 (21.5%) | 0 | 160 | 0 |
| **Inhaled LABA/ICS** | **Salmeterol/Fluticasone (fixed combination)** | 0-1 | 66 | 66 (100.0%) | 35 | 0 | 31 | 24 | 24 (100.0%) | 10 | 0 | 14 |
|  |  | 2-5 | 1,570 | 573 (36.5%) | 211 | 284 | 78 | 950 | 381 (40.1%) | 112 | 216 | 53 |
|  |  | 6-11 | 8,242 | 1,025 (12.4%) | 0 | 1,025 | 0 | 4,354 | 628 (14.4%) | 0 | 628 | 0 |
|  |  | 12-14 | 4,078 | 473 (11.6%) | 0 | 473 | 0 | 2,050 | 255 (12.4%) | 0 | 255 | 0 |
|  |  | 15-18 | 3,597 | 395 (11.0%) | 0 | 395 | 0 | 2,669 | 458 (17.2%) | 0 | 458 | 0 |
|  | **Formoterol/Beclomethasone (fixed combination)** | 0-1 | 1 | 1 (100.0%) | 0 | 0 | 1 | 1 | 1 (100.0%) | 0 | 0 | 1 |
|  |  | 2-5 | 13 | 13 (100.0%) | 9 | 0 | 4 | 6 | 6 (100.0%) | 2 | 0 | 4 |
|  |  | 6-11 | 111 | 28 (25.2%) | 0 | 28 | 0 | 72 | 24 (33.3%) | 0 | 24 | 0 |
|  |  | 12-14 | 255 | 88 (34.5%) | 0 | 88 | 0 | 239 | 68 (28.5%) | 0 | 68 | 0 |
|  |  | 15-18 | 823 | 255 (31.0%) | 0 | 255 | 0 | 994 | 364 (36.6%) | 0 | 364 | 0 |
|  | **Formoterol/Budesonide (fixed combination)** | 0-1 | 1 | 1 (100.0%) | 1 | 0 | 0 | 1 | 1 (100.0%) | 0 | 0 | 1 |
|  |  | 2-5 | 125 | 125 (100.0%) | 86 | 0 | 39 | 45 | 45 (100.0%) | 26 | 0 | 19 |
|  |  | 6-11 | 3,252 | 549 (16.9%) | 0 | 549 | 0 | 1,664 | 286 (17.2%) | 0 | 286 | 0 |
|  |  | 12-14 | 2,461 | 327 (13.3%) | 0 | 327 | 0 | 1,315 | 223 (17.0%) | 0 | 223 | 0 |
|  |  | 15-18 | 2,553 | 449 (17.6%) | 0 | 449 | 0 | 2,416 | 578 (23.9%) | 0 | 578 | 0 |
| **Inhaled SAMA** | **Ipratropium** | 0-1 | 4,017 | 2,431 (60.5%) | 0 | 2,431 | 0 | 2,360 | 1,550 (65.7%) | 0 | 1,550 | 0 |
|  |  | 2-5 | 5,666 | 2,754 (48.6%) | 0 | 2,754 | 0 | 3,505 | 1,879 (53.6%) | 0 | 1,879 | 0 |
|  |  | 6-11 | 3,224 | 1,195 (37.1%) | 0 | 1,195 | 0 | 1,851 | 716 (38.7%) | 0 | 716 | 0 |
|  |  | 12-14 | 478 | 123 (25.7%) | 0 | 123 | 0 | 274 | 87 (31.8%) | 0 | 87 | 0 |
|  |  | 15-18 | 257 | 102 (39.7%) | 0 | 102 | 0 | 190 | 73 (38.4%) | 0 | 73 | 0 |
| **Inhaled LAMA** | **Tiotropium** | 0-1 | 0 | 0 | 0 | 0 | 0 | 0 | 0 | 0 | 0 | 0 |
|  |  | 2-5 | 1 | 1 (100.0%) | 0 | 0 | 1 | 0 | 0 | 0 | 0 | 0 |
|  |  | 6-11 | 7 | 7 (100.0%) | 0 | 0 | 7 | 8 | 8 (100.0%) | 1 | 0 | 7 |
|  |  | 12-14 | 14 | 14 (100.0%) | 0 | 0 | 14 | 8 | 8 (100.0%) | 2 | 0 | 6 |
|  |  | 15-18 | 20 | 20 (100.0%) | 3 | 4 | 13 | 39 | 38 (97.4%) | 3 | 7 | 28 |
| **ICS** | **Budesonide** | 0-1 | 3,197 | 183 (5.7%) | 0 | 183 | 0 | 1,797 | 107 (6.0%) | 0 | 107 | 0 |
|  |  | 2-5 | 7,762 | 517 (6.7%) | 0 | 517 | 0 | 4,906 | 380 (7.7%) | 0 | 380 | 0 |
|  |  | 6-11 | 8,872 | 544 (6.1%) | 0 | 544 | 0 | 4,919 | 359 (7.3%) | 0 | 359 | 0 |
|  |  | 12-14 | 2,822 | 186 (6.6%) | 0 | 186 | 0 | 1,692 | 155 (9.2%) | 0 | 155 | 0 |
|  |  | 15-18 | 3,034 | 293 (9.7%) | 0 | 293 | 0 | 3,066 | 442 (14.4%) | 0 | 442 | 0 |
|  | **Beclomethasone** | 0-1 | 1,876 | 43 (2.3%) | 0 | 43 | 0 | 985 | 32 (3.3%) | 0 | 32 | 0 |
|  |  | 2-5 | 4,568 | 282 (6.2%) | 0 | 282 | 0 | 2,914 | 195 (6.7%) | 0 | 195 | 0 |
|  |  | 6-11 | 5,171 | 405 (7.8%) | 0 | 405 | 0 | 3,213 | 316 (9.8%) | 0 | 316 | 0 |
|  |  | 12-14 | 1,636 | 150 (9.2%) | 0 | 150 | 0 | 1,063 | 141 (13.3%) | 0 | 141 | 0 |
|  |  | 15-18 | 1,253 | 152 (12.1%) | 0 | 152 | 0 | 1,506 | 206 (13.7%) | 0 | 206 | 0 |
|  | **Fluticasone** | 0-1 | 720 | 720 (100.0%) | 411 | 0 | 309 | 279 | 279 (100.0%) | 157 | 0 | 122 |
|  |  | 2-5 | 3,295 | 1,689 (51.3%) | 850 | 476 | 363 | 2,115 | 1,134 (53.6%) | 499 | 356 | 279 |
|  |  | 6-11 | 4,948 | 700 (14.1%) | 0 | 700 | 0 | 2,825 | 430 (15.2%) | 0 | 430 | 0 |
|  |  | 12-14 | 1,240 | 150 (12.1%) | 0 | 150 | 0 | 597 | 86 (14.4%) | 0 | 86 | 0 |
|  |  | 15-18 | 676 | 107 (15.8%) | 0 | 107 | 0 | 402 | 65 (16.2%) | 0 | 65 | 0 |
|  | **Ciclesonide** | 0-1 | 3 | 3 (100.0%) | 1 | 0 | 2 | 4 | 4 (100.0%) | 1 | 0 | 3 |
|  |  | 2-5 | 3 | 3 (100.0%) | 3 | 0 | 0 | 3 | 3 (100.0%) | 1 | 0 | 2 |
|  |  | 6-11 | 23 | 23 (100.0%) | 18 | 0 | 5 | 14 | 14 (100.0%) | 11 | 0 | 3 |
|  |  | 12-14 | 57 | 8 (14.0%) | 0 | 8 | 0 | 47 | 11 (23.4%) | 0 | 11 | 0 |
|  |  | 15-18 | 63 | 11 (17.5%) | 0 | 11 | 0 | 109 | 30 (27.5%) | 0 | 30 | 0 |
| **Oral B2A** | **Salbutamol** | 0-1 | 4,359 | 1,098 (25.2%) | 0 | 1,098 | 0 | 2,920 | 754 (25.8%) | 0 | 754 | 0 |
|  |  | 2-5 | 5,128 | 1,457 (28.4%) | 0 | 1,457 | 0 | 4,012 | 1,212 (30.2%) | 0 | 1,212 | 0 |
|  |  | 6-11 | 1,482 | 490 (33.1%) | 0 | 490 | 0 | 1,162 | 381 (32.8%) | 0 | 381 | 0 |
|  |  | 12-14 | 192 | 65 (33.9%) | 0 | 65 | 0 | 103 | 48 (46.6%) | 0 | 48 | 0 |
|  |  | 15-18 | 45 | 16 (35.6%) | 0 | 16 | 0 | 72 | 23 (31.9%) | 0 | 23 | 0 |
|  | **Terbutaline** | 0-1 | 1,292 | 262 (20.3%) | 0 | 262 | 0 | 845 | 200 (23.7%) | 0 | 200 | 0 |
|  |  | 2-5 | 1,760 | 515 (29.3%) | 0 | 515 | 0 | 1,384 | 446 (32.2%) | 0 | 446 | 0 |
|  |  | 6-11 | 815 | 266 (32.6%) | 0 | 266 | 0 | 644 | 254 (39.4%) | 0 | 254 | 0 |
|  |  | 12-14 | 72 | 26 (36.1%) | 0 | 26 | 0 | 46 | 12 (26.1%) | 0 | 12 | 0 |
|  |  | 15-18 | 43 | 12 (27.9%) | 0 | 12 | 0 | 39 | 19 (48.7%) | 0 | 19 | 0 |
|  | **Tulobuterol** | 0-1 | 102 | 31 (30.4%) | 17 | 9 | 5 | 72 | 32 (44.4%) | 15 | 16 | 1 |
|  |  | 2-5 | 348 | 89 (25.6%) | 0 | 89 | 0 | 273 | 67 (24.5%) | 0 | 67 | 0 |
|  |  | 6-11 | 217 | 54 (24.9%) | 0 | 54 | 0 | 138 | 41 (29.7%) | 0 | 41 | 0 |
|  |  | 12-14 | 22 | 4 (18.2%) | 0 | 4 | 0 | 17 | 7 (41.2%) | 0 | 7 | 0 |
|  |  | 15-18 | 6 | 1 (16.7%) | 0 | 1 | 0 | 6 | 4 (66.7%) | 0 | 4 | 0 |
|  | **Clenbuterol** | 0-1 | 8 | 6 (75.0%) | 0 | 6 | 0 | 1 | 0 | 0 | 0 | 0 |
|  |  | 2-5 | 16 | 6 (37.5%) | 0 | 6 | 0 | 8 | 5 (62.5%) | 0 | 5 | 0 |
|  |  | 6-11 | 19 | 11 (57.9%) | 0 | 11 | 0 | 9 | 2 (22.2%) | 0 | 2 | 0 |
|  |  | 12-14 | 7 | 4 (57.1%) | 0 | 4 | 0 | 6 | 4 (66.7%) | 0 | 4 | 0 |
|  |  | 15-18 | 8 | 2 (25.0%) | 0 | 2 | 0 | 31 | 27 (87.1%) | 0 | 27 | 0 |
| **Oral B2A combination** | **Clenbuterol/Ambroxol (fixed combination)** | 0-1 | 13,684 | 2,021 (14.8%) | 0 | 2,021 | 0 | 9,169 | 1,440 (15.7%) | 0 | 1,440 | 0 |
|  |  | 2-5 | 22,014 | 4,654 (21.1%) | 0 | 4,654 | 0 | 17,249 | 3,711 (21.5%) | 0 | 3,711 | 0 |
|  |  | 6-11 | 12,573 | 2,906 (23.1%) | 0 | 2,906 | 0 | 8,922 | 2,287 (25.6%) | 0 | 2,287 | 0 |
|  |  | 12-14 | 2,485 | 562 (22.6%) | 0 | 562 | 0 | 1,516 | 433 (28.6%) | 0 | 433 | 0 |
|  |  | 15-18 | 1,912 | 407 (21.3%) | 0 | 407 | 0 | 1,861 | 476 (25.6%) | 0 | 476 | 0 |
| **Others** | **Theophylline** | 0-1 | 36 | 33 (91.7%) | 5 | 11 | 17 | 20 | 14 (70.0%) | 2 | 4 | 8 |
|  |  | 2-5 | 70 | 28 (40.0%) | 0 | 28 | 0 | 54 | 28 (51.9%) | 0 | 28 | 0 |
|  |  | 6-11 | 168 | 48 (28.6%) | 0 | 48 | 0 | 105 | 34 (32.4%) | 0 | 34 | 0 |
|  |  | 12-14 | 136 | 41 (30.1%) | 0 | 41 | 0 | 72 | 24 (33.3%) | 0 | 24 | 0 |
|  |  | 15-18 | 230 | 75 (32.6%) | 0 | 75 | 0 | 293 | 126 (43.0%) | 0 | 126 | 0 |
|  | **Montelukast** | 0-1 | 2,207 | 1,593 (72.2%) | 209 | 944 | 440 | 1,081 | 850 (78.6%) | 95 | 504 | 251 |
|  |  | 2-5 | 7,412 | 3,262 (44.0%) | 0 | 3262 | 0 | 4,610 | 2,340 (50.8%) | 0 | 2340 | 0 |
|  |  | 6-11 | 7,796 | 1,889 (24.2%) | 0 | 1889 | 0 | 4,497 | 1,374 (30.6%) | 0 | 1374 | 0 |
|  |  | 12-14 | 2,107 | 473 (22.4%) | 0 | 473 | 0 | 1,171 | 317 (27.1%) | 0 | 317 | 0 |
|  |  | 15-18 | 1,336 | 313 (23.4%) | 0 | 313 | 0 | 1,284 | 415 (32.3%) | 0 | 415 | 0 |
|  | **Cromoglicic acid** | 0-1 | 303 | 303 (100.0%) | 71 | 0 | 232 | 220 | 220 (100.0%) | 39 | 0 | 181 |
|  |  | 2-5 | 1,187 | 801 (67.5%) | 0 | 801 | 0 | 891 | 657 (73.7%) | 0 | 657 | 0 |
|  |  | 6-11 | 1,358 | 686 (50.5%) | 0 | 686 | 0 | 830 | 455 (54.8%) | 0 | 455 | 0 |
|  |  | 12-14 | 148 | 51 (34.5%) | 0 | 51 | 0 | 68 | 30 (44.1%) | 0 | 30 | 0 |
|  |  | 15-18 | 46 | 20 (43.5%) | 0 | 20 | 0 | 36 | 24 (66.7%) | 0 | 24 | 0 |
