# Supplementary material for: Utilisation and Off-Label Prescriptions of Respiratory Drugs in Children
Source: PLoS One. 2014 Sep 2;9(9):e105110. doi: 10.1371/journal.pone.0105110 (PMC4152124; doi:10.1371/journal.pone.0105110)
Supplement: Table S5 — Number and proportion of off-label prescriptions for the years 2004 to 2008. SABA: Short-acting beta-2-agonist, CGA: Cromoglicic Acid, LABA: Long-acting beta-2-agonist, ICS: Inhaled corticosteroid, SAMA: Short-acting muscarinic antagonist, LAMA: Long-acting muscarinic antagonist, B2A: Beta-2-agonist, n.a.: not applicable. (DOC) [file pone.0105110.s005.doc]

Table S5: Number and proportion of off-label prescriptions for the years 2004 to 2008. SABA: Short-acting beta-2-agonist, CGA: Cromoglicic acid, LABA: Long-acting beta-2-agonist, ICS: Inhaled corticosteroid, SAMA: Short-acting muscarinic antagonist, LAMA: Long-acting muscarinic antagonist, B2A: Beta-2-agonist, n.a.: not applicable.

| **Compound class** | **Compound** | **2004** | **2005** | **2006** | **2007** | **2008** | **Delta 2004 vs. 2008** |
| --- | --- | --- | --- | --- | --- | --- | --- |
| **Inhaled SABA** | **Salbutamol** | 45,243 (37.5%) | 67,644 (46.7%) | 66,502 (45.9%) | 73,357 (45.6%) | 67,084 (42.0%) | +21,841 (+48.3%) |
|  | **Fenoterol** | 530 (28.4%) | 485 (27.0%) | 458 (26.8%) | 508 (28.3%) | 383 (26.4%) | -147 (-27.7%) |
|  | **Terbutaline** | 394 (45.2%) | 222 (37.0%) | 43 (13.6%) | 42 (17.6%) | 34 (18.5%) | -360 (-91.4%) |
| **Inhaled SABA combination** | **Fenoterol/Ipratropium (fixed combination)** | 3,407 (36.7%) | 3,875 (50.5%) | 2,553 (44.9%) | 2,277 (45.7%) | 1,722 (43.1%) | -1,685 (-49.5%) |
|  | **Reproterol/CGA (fixed combination)** | 6,681 (34.3%) | 5,882 (32.7%) | 4,070 (30.0%) | 3,710 (31.0%) | 2,538 (29.1%) | -4,143 (-62.0%) |
| **Inhaled LABA** | **Salmeterol** | 392 (19.9%) | 294 (20.4%) | 137 (14.8%) | 149 (19.9%) | 86 (16.5%) | - 306 (-78.1%) |
|  | **Formoterol** | 1,008 (19.1%) | 1,336 (22.7%) | 1,177 (20.9%) | 1,107 (19.7%) | 839 (17.0%) | -169 (-16.8%) |
| **Inhaled LABA/ICS** | **Salmeterol/Fluticasone (fixed combination)** | 4,196 (18.6%) | 5,584 (20.8%) | 4,790 (17.4%) | 4,792 (17.2%) | 4,278 (15.5%) | +82 (+2.0%) |
|  | **Formoterol/Budesonide (fixed combination)** | 2,362 (23.2%) | 3,435 (24.9%) | 3,144 (21.8%) | 3,077 (20.8%) | 2,584 (18.7%) | +222 (+9.4%) |
|  | **Formoterol/Beclomethasone (fixed combination)** | n.a. | n.a. | 55 (37.2%) | 654 (36.2%) | 848 (33.7%) | n.a. |
| **Inhaled SAMA** | **Ipratropium** | 11,297 (41.3%) | 15,838 (53.7%) | 13,205 (53.1%) | 12,748 (52.9%) | 10,910 (50.0%) | -387 (-3.4%) |
| **Inhaled LAMA** | **Tiotropium** | 145 (96.7%) | 190 (96.0%) | 112 (93.3%) | 92 (98.9%) | 96 (99.0%) | -49 (-33.8%) |
| **ICS** | **Budesonide** | 2,566 (6.3%) | 3,223 (6.6%) | 3,145 (7.3%) | 3,216 (7.3%) | 3,166 (7.5%) | +600 (+23.4%) |
|  | **Beclomethasone** | 841 (8.1%) | 1,094 (9.2%) | 1,596 (8.7%) | 1,807 (8.1%) | 1,922 (7.9%) | +1,081 (+128.5%) |
|  | **Fluticasone** | 4,854 (30.4%) | 6,158 (36.0%) | 6,024 (34.2%) | 5,946 (33.8%) | 5,360 (31.4%) | +506 (+10.4%) |
|  | **Ciclesonide** | n.a. | 230 (43.1%) | 524 (44.1%) | 709 (41.8%) | 110 (33,7%) | n.a. |
| **Oral B2A** | **Salbutamol** | 122 (21.7%) | 115 (20.3%) | 51 (14.2%) | 696 (26.7%) | 5,544 (28.5%) | +5,422 (+4,444.3 %) |
|  | **Terbutaline** | 7,086 (28.4%) | 9,647 (30.3%) | 8,678 (27.6%) | 9,549 (28.0%) | 2,012 (29.0%) | -5,074 (-71.6%) |
|  | **Tulobuterol** | 893 (28.3%) | 1,180 (33.4%) | 660 (30.3%) | 486 (25.6%) | 330 (27.5%) | -563 (-63.0%) |
|  | **Clenbuterol** | 1,385 (33.9%) | 1,414 (34.9%) | 1,250 (34.1%) | 268 (28.4%) | 67 (59.3%) | -1,318 (-95.2%) |
| **Oral B2A combination** | **Clenbuterol/Ambroxol (fixed combination)** | 25,093 (19.5%) | 30,941 (20.8%) | 21,239 (19.6%) | 20,875 (20.2%) | 18,897 (20.7%) | -6,196 (-24.7%) |
| **Others** | **Theophylline** | 1,163 (38.5%) | 1,192 (42.0%) | 768 (41.4%) | 611 (40.3%) | 451 (38.1%) | -712 (-61.2%) |
|  | **Montelukast** | 8,551 (39.6%) | 12,665 (41.6%) | 13,747 (39.8%) | 16,704 (42.3%) | 12,826 (38.3%) | +4,275 (+50.0%) |
|  | **Cromoglicic acid** | 13,442 (61.2%) | 10,897 (62.4%) | 6,548 (61.1%) | 5,051 (63.5%) | 3,247 (63.8%) | -10,195 (-75.8%) |
