# Supplementary material for: Utilisation and Off-Label Prescriptions of Respiratory Drugs in Children
Source: PLoS One. 2014 Sep 2;9(9):e105110. doi: 10.1371/journal.pone.0105110 (PMC4152124; doi:10.1371/journal.pone.0105110)
Supplement: Table S6 — Comparison of the proportion of off-label usage due to indication in several European countries. SABA: Short-acting beta-2-agonist, LABA: Long-acting beta-2-agonist, ICS: Inhaled corticosteroid, SAMA: Short-acting muscarinic antagonist. (DOC) [file pone.0105110.s006.doc]

Table S6: Comparison of the proportion of off-label usage due to indication in several European countries. SABA: Short-acting beta-2-agonist, LABA: Long-acting beta-2-agonist, ICS: Inhaled corticosteroid, SAMA: Short-acting muscarinic antagonist.

| **Compound class** | **Compound** | **Proportion of off-label use due to indication [%]** | | | | |
| --- | --- | --- | --- | --- | --- | --- |
|  |  | **Netherlands** | **Italy*** | **Italy** | **UK** | **Germany (Schmiedl et al.)** |
| **Inhaled SABA** | **Salbutamol** | 32.8 | 46.2 | - | - | 42.0 |
|  | **Terbutaline** | 30.7 | - | - | 23.5 | 18.5 |
| **Inhaled SABA combination** | **Fenoterol/Ipratropium (fixed combination)** | 63.0 | - | - | - | 43.1 |
| **Inhaled LABA** | **Salmeterol** | 19.0 | - | - | 12.1 | 14.8 |
|  | **Formoterol** | 16.4 | - | - | - | 15.2 |
| **Inhaled LABA/ICS** | **Salmeterol/Fluticasone (fixed combination)** | 24.4 | 29.3 | 31.6 | - | 14.2 |
|  | **Formoterol/Budesonide (fixed combination)** | 28.4 | - | 29.1 | 10.8 | 17.9 |
| **Inhaled SAMA** | **Ipratropium** | - | 80.1 | 80.1 | 74.2 | 50.0 |
| **ICS** | **Budesonide** | - | 78.0 | 80.0 | 21.0 | 7.5 |
|  | **Beclomethasone** | 27.2 | 66.8 | - | 25.8 | 7.9 |
|  | **Fluticasone** | - | 46.3 | - | 18.7 | 20.1 |
| **Others** | **Montelukast** | 16.0 | 62.3 | 36.1 | - | 37.4 |
|  | **Cromoglicic acid** | 26.3 | - | 51.3 | 34.7 | 61.7 |

* Exclusive missing diagnoses.
